# PDMS and DLC‐coated unidirectional valves for artificial urinary sphincters: Opening performance after 126 days of immersion in urine

**DOI:** 10.1002/jbm.b.34961

**Published:** 2021-11-02

**Authors:** Tommaso Mazzocchi, Gioia Lucarini, Irene Roehrer, Arianna Menciassi, Leonardo Ricotti

**Affiliations:** ^1^ The BioRobotics Institute, Scuola Superiore Sant'Anna Pisa Italy; ^2^ Department of Excellence in Robotics & AI Scuola Superiore Sant'Anna Pisa Italy

**Keywords:** artificial sphincter, diamond‐like carbon, polydimethylsiloxane, polymeric valve, urinary encrustation, urine‐resistant coating

## Abstract

In this work, unidirectional valves made of bare polydimethylsiloxane (PDMS) and PDMS provided with a micrometric diamond‐like carbon (DLC) coating were fabricated and characterized, in terms of surface properties and opening pressure. The valve performance was also tested over 1250 repeated cycles of opening/closure in water, finding a slight decrease in the opening pressure after such cycles (10%) for the PDMS valves, while almost no variation for the PDMS + DLC ones. The valves were then immersed in urine for 126 days, evaluating the formation of encrustations and the trend of the opening pressure over time. Results showed that PDMS valves were featured by a thin layer of encrustations after 126 days, but the overall encrustation level was much smaller than the one shown by PDMS in static conditions. Furthermore, the opening pressure was almost not affected by such a thin layer of crystals. DLC‐coated valves showed even less encrustations at the same time‐point, with no significant loss of performance over time, although they were featured by a higher variability. These results suggest that most encrustations can be removed by the mechanical action of the valve during daily openings/closures. Such a self‐cleaning behavior with respect to a static condition opens exciting scenarios for the long‐term functionality of mobile devices operating in the urinary environment.

## 1 INTRODUCTION

Urinary incontinence (UI) is a frustrating and embarrassing condition that has a profound influence on patients' quality of life as well as on health care costs.^1^ The number of people who experience UI worldwide significantly increased in recent years, rising from 348 to 386 million between 2008 and 2013^2^ and overcoming 400 million in 2017.^3^ The related costs are enormous: The global incontinence care products and devices' market was valued at US$ 9,861.3 million in 2014 and at US$ 13,747.7 million in 2019, with a CAGR of 6.9% from 2015 to 2019.^4^Depending on the severity level, different solutions have been adopted to face UI.^5^ Recently, especially for severe stress UI, artificial urinary sphincters (AUS) proved to be promising solutions. AUS can be classified in extra‐urethral and endo‐urethral ones. The extra‐urethral AUS constitute the most common solution commercially available at present. In particular, the AMS 800 (Boston Scientific) represents the gold standard. This device is installed around the urethra through an invasive surgical operation and restores continence by circumferentially compressing it.^6^, ^7^

On the other hand, endo‐urethral AUS consist of miniaturized devices, featured by significantly smaller invasiveness, placed inside the urethral canal by an endo‐luminal procedure with standard urinary tools under local or total anesthesia or by self‐insertion by the patient. Only a few endo‐urethral solutions are currently on the market, such as the Reliance System (UroMed Inc.)^8^ and the FemSoft Insert (Rochester Medical Corp.).^9^

Recently, the authors proposed an innovative endo‐urethral AUS based on a unidirectional polymeric valve and a magnetic activation/deactivation system.^10^ Such a device can be inserted/removed in a minimally invasive fashion such that it does not alter the body scheme and can be applied to both women and men. Bench tests and ex vivo tests on a human cadaver demonstrated that the device was able to restore continence and to allow urination when desired. However, efficient long‐term functionality after implantation in patients still has to be demonstrated. One of the most critical risks affecting endo‐urethral AUS is related to their permanence in a rather hostile environment, being in continuous contact with the urine. Encrustations and infections, indeed, still represent an open issue for medical devices to be employed in the urinary system: Urine alters the device properties and functionality, raising the need for frequent replacements.^11^ Regarding the AUS reported in Reference 10, the main component subjected to a risk of functional failure due to encrustations is the polymeric valve, made of polydimethylsiloxane (PDMS).^12^ Anyhow, there are many other endo‐urethral devices based on silicone.^9^ Thus, assessing the functionality over time of silicone components (the PDMS valve in this case) would provide important hints on the possible long‐term stability of these devices in the urinary system.

Cardona et al. investigated the behavior of PDMS samples in terms of formation of encrustations, also testing the effect of urine‐resistant coatings (nanostructured diamond‐like carbon [DLC], MoS_2_ nanoparticles, and WS_2_ nanoparticles) in comparison with the bare polymer.^13^ However, in this study, the materials were maintained in static conditions (without opening the valve) for 4 weeks. At present, no studies focused on the dynamic performance (in terms of opening pressure, which is critical for guaranteeing AUS function) of polymeric valves after several weeks of contact with the urine. To fill this gap, this study compares the performance of bare PDMS and DLC‐coated unidirectional valves for AUS, both in terms of fatigue behavior and opening pressure, over 126 days of immersion in different types of urine with daily openings. We hypothesize that such a dynamic condition (the periodic opening of the valve and the flow of urine through it) prevents the risk that encrustations block the valve, hampering its functionality.

In this work, we demonstrated that PDMS‐based valves, whose constitutive material typically undergoes massive encrustation after a few weeks, in both sterile and urease‐enriched urine,^13^ have unchanged performance over time, in terms of opening pressure. The polymeric valves made of such a material, in this study, outperformed even the long‐term indwelling catheters (that after 12 weeks fail), demonstrating that the periodical mechanical opening of the valve (multiple times per day) is effective in “resetting” the chain of events that bring from the first small crystalline deposit to the large struvite, apatite, or calcium oxalate crystals, not giving them the time they need to form. Being fully effective over 4 months, it is reasonable assuming that this “mechanically induced progression‐breaking effect” of urinary encrustations would be maintained in the following months, suggesting long‐term stability of the device over several months/years.

## 2 RESULTS

### 2.1 Fabrication and surface characterization

**FIGURE 1:**
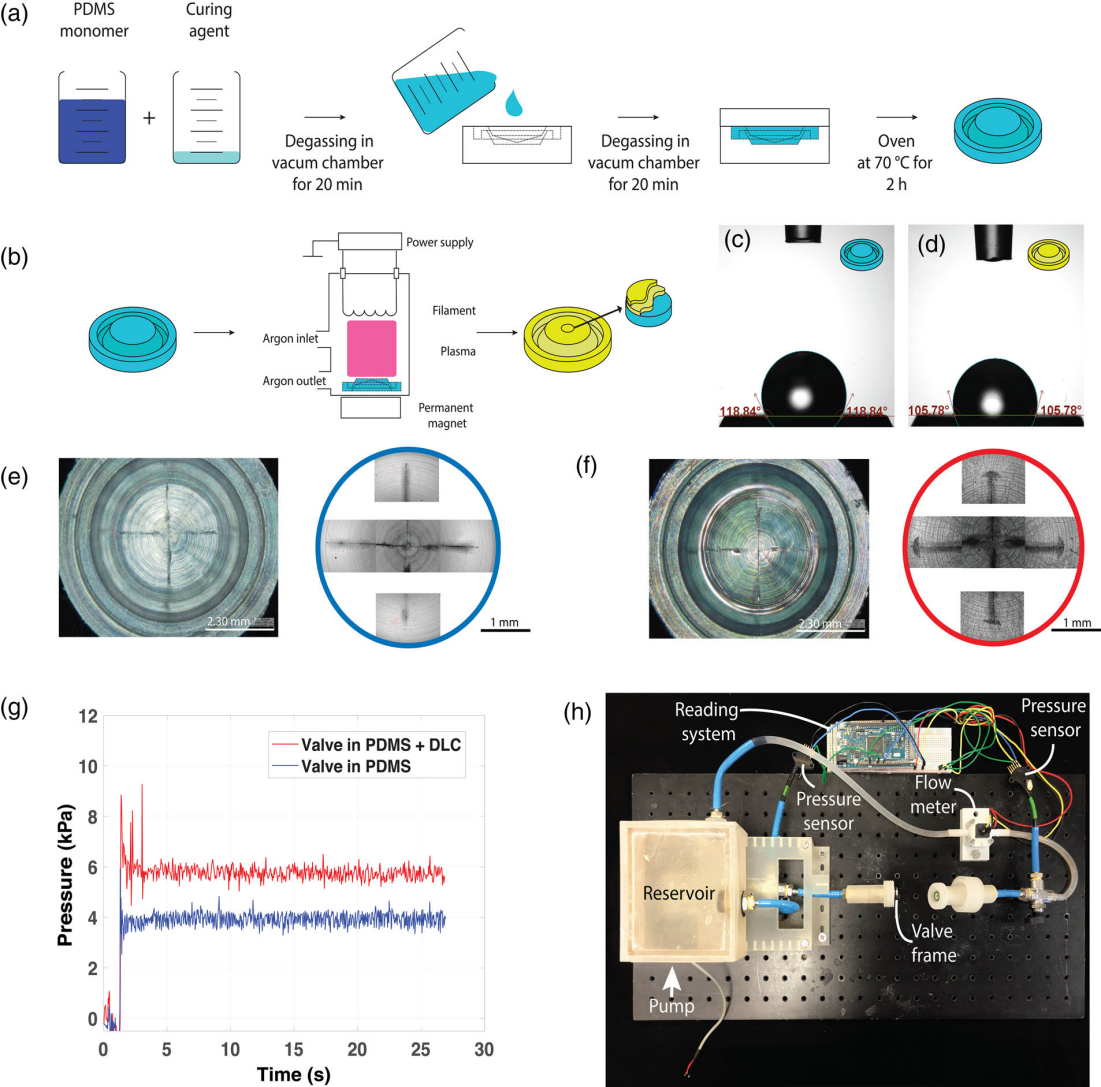
Fabrication and testing of the valves. (A) Scheme of fabrication for the PDMS valves. (B) Scheme of the procedure used to provide PDMS valves with a DLC coating (blue is PDMS, yellow is DLC coating). The two types of valves have been characterized in terms of contact angles (C) and (D), surface morphology and roughness (E) and (F), and opening pressure (G), through a custom setup shown in (H). DLC, Diamond‐like carbon; PDMS, polydimethylsiloxane

Polymeric PDMS valves were successfully fabricated through a molding procedure (Figure 1A). Some valves were then coated with DLC (Figure 1B). In fact, DLC has been recently proposed as a coating for urinary stents, especially those made of polyurethane,^14^, ^15^ for reducing crystalline fouling.^16^ It is thus interesting to verify its behavior in dynamic conditions, with respect to the bare PDMS. Plasma‐enhanced chemical vapor deposition (PE‐CVD; T < 180°C) was first used to deposit a first soft DLC layer (with a relatively low number of sp^3^ bonds), aimed at improving the adhesion on PDMS substrates. Such a layer had a thickness of ∼50 nm. On top of it, a hard‐DLC layer (with a higher number of sp^3^ bonds, to guarantee chemical inertia) was fabricated using the same technique. The thickness of the second layer was ∼50 nm too. Such a double‐layer structure avoided an abrupt transition of elastic modulus between the substrate and the coating, thus preventing the formation of cracks that could compromise the long‐term resistance to urine. The coating of DLC layer was performed by NCD Technologies Inc.^17^

PDMS and PDMS+DLC valves were then characterized in terms of contact angle (Figure 1C,D), surface morphology (Figure 1E), and roughness (Figure 1F). Opening pressure was also evaluated through a custom setup (Figure 1G). The opening pressure, indeed, is a crucial property of unidirectional valve that determines their functionality: An alteration of the opening pressure implies that the artificial sphincter is activated (and the patient urinates) at a different intravesical pressure.

Results showed a hydrophobic behavior of both valve types (contact angle values were *θ*_PDMS_ = 119.0 ± 0.2°; *θ*_DLC_ = 108 ± 3°). Optical profilometric analyses revealed a rather flat surface for both valve types, with a peculiar microstructure in the case of DLC‐coated valves (Figure S1). The surface roughness values were respectively equal to *R*_PDMS_ ≅ 0.8 ± 0.4 μm and *R*_DLC_ ≅ 2.7 ± 0.9 μm. The valve opening pressures were measured through the setup shown in Figure 1G (more details are reported in Section 4). Results (Figure 1H) showed that the initial opening pressure values resulted different: *P*_opening,PDMS_ = 4.2 ± 0.1 kPa; *P*_opening,DLC_ = 5.5 ± 0.7 kPa.

### 2.2 Fatigue tests

**FIGURE 2:**
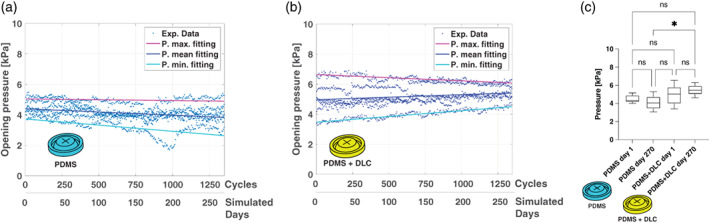
Fatigue test: opening pressure versus time for PDMS valves (a) and PDMS + DLC valve (b). (c) Shows a parametric comparative analysis Normality tests (D'Agostino‐Pearson) were performed on all experimental data to assess the data distribution, obtaining not normal data. Results were expressed as mean ± SD. Data analysis performed by applying the Kruskal–Wallis with Dunn's post hoc tests were adopted for multiple comparisons. Statistical analyses were carried out using GraphPad Prism (v 8.0.2). DLC, Diamond‐like carbon; PDMS, polydimethylsiloxane. The significance threshold was set at 5% (*p < .05)

Fatigue tests were performed through the setup shown in Figure 1H. These tests allowed to evaluate the capability of the valves to keep their performances over time and to check the adhesion and the stability of the DLC coating after repeated opening cycles. The valves (three samples for each type) were subjected to 1350 cycles of opening/closure, corresponding to a simulated lifetime of 9 months (assuming five urinations per day), by flowing 200 ml of water at each cycle and measuring the valve opening pressure. Results are shown in Figure 2. The trend of the valve opening pressures shows that the performance remains almost unvaried over time in both cases: The valves in PDMS showed a slight decrease in the opening pressure value, which presented a loss of ~0.56 kPa (corresponding to 12%) in the simulated 9 months, while the vale in PDMS + DLC showed: (i) a slight increment of the opening pressure corresponding to 11% and (ii) a significant reduction of the deviation standard corresponding to 44%. The performances recorded in terms of opening pressure at Cycle 1 and Cycle 270 for each valve (PDMS and PDMS + DLC) did not show statistically significant differences (Figure 2C).

**FIGURE 3:**
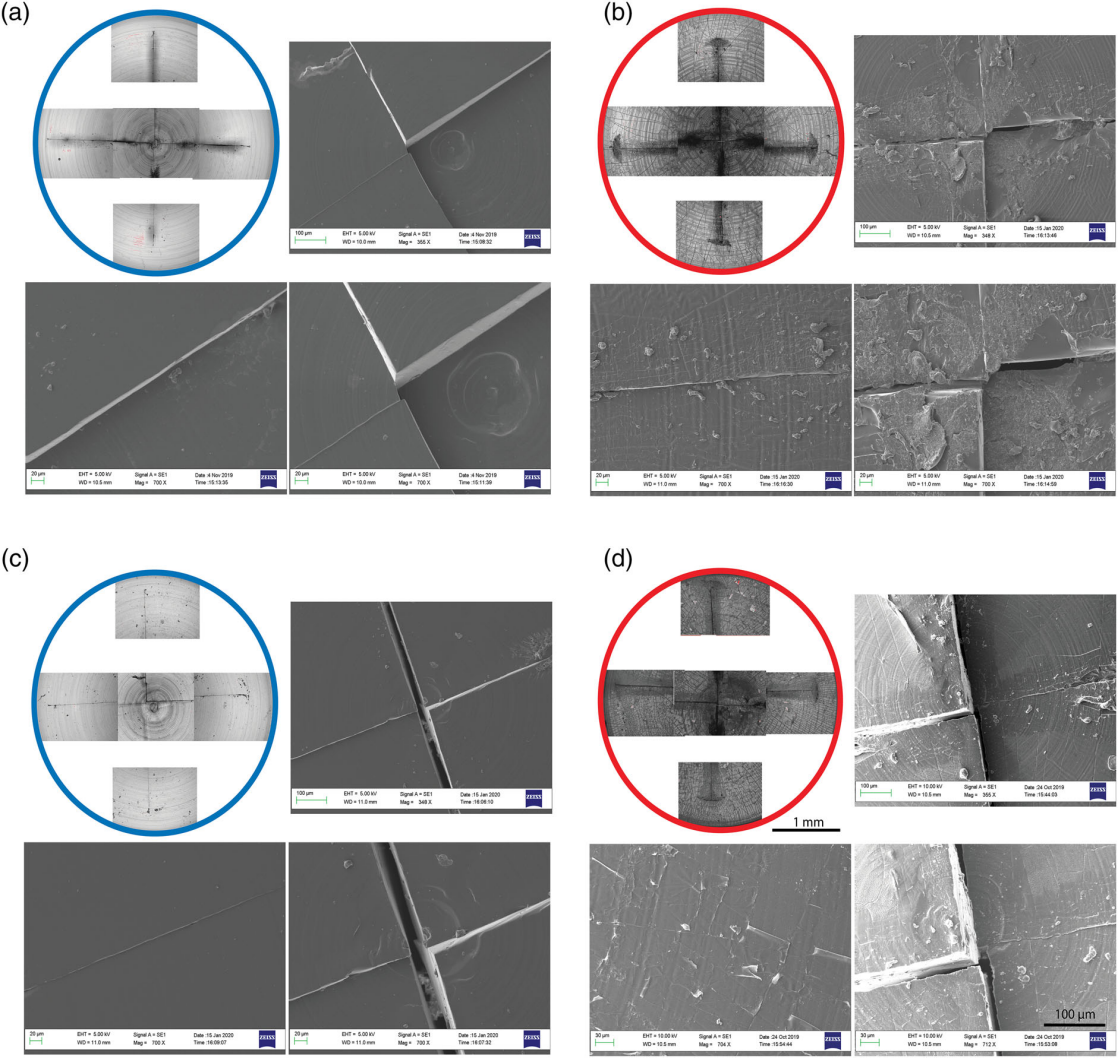
Fatigue test: SEM images. Comparison between PDMS and PDMS + DLC valves before and after continuous working cycles in water. Here, a zoom view of the valves in the center (right, different zooms) and in the peripheral area (left) is reported. DLC, Diamond‐like carbon; PDMS, polydimethylsiloxane; SEM, scanning electron microscopy

The surface of the different valves was qualitatively analyzed through optical profilometry and scanning electron microscopy (SEM; Figure 3). The PDMS surface showed no visible alterations after 1350 stress cycles, with respect to the non‐stressed samples. As regard the PDMS + DLC valves, they were featured, in the initial state, by a uniform coverage of the substrate, characterized by small cracks running along the entire valve surface. Following the fatigue test, the initial cracks become deeper and more evident. The roughness values passed from *R*_DLC_ = 2.73 ± 0.9 μm to *R*_DLC_fatigue_ = 5.23 ± 3.2 μm. The DLC layer appeared, in certain points, slightly exfoliated. However, overall, it still covered mostly the substrate surface. Therefore, the DLC coating resulted in capable of resisting valve movements.

### 2.3 Behavior after immersion in urine

This experiment aimed at evaluating the resistance of the PDMS and PDMS + DLC valves to the formation of urinary encrustations and their performance (in terms of opening pressure) over time in urine when the valves were opened five times per day with the setup shown in Figure 1H by flowing 200 ml of water through the valve. This mimicked the urination frequency of a standard subject. The valves were incubated for 16 weeks (with experimental time‐points at 2 and 8 weeks, for SEM imaging) in artificial urine solutions. Because the urine parameters such as pH and protein content are subjected to a considerable variability depending on the subject and his/her overall conditions, three different urine solutions were considered. This allowed testing standard as well as altered precipitation conditions in which the encrustations are more favored.^13^, ^18^ The used formulations were (i) sterile artificial urine (Sterile); (ii) artificial urine with the addition of the urease enzyme, to simulate the presence of a bacterial infection (Urease); and (iii) artificial urine with the addition of the urease enzyme and the albumin protein (Albumin) to evaluate the role of the protein component in the encrustation process.

**FIGURE 4:**
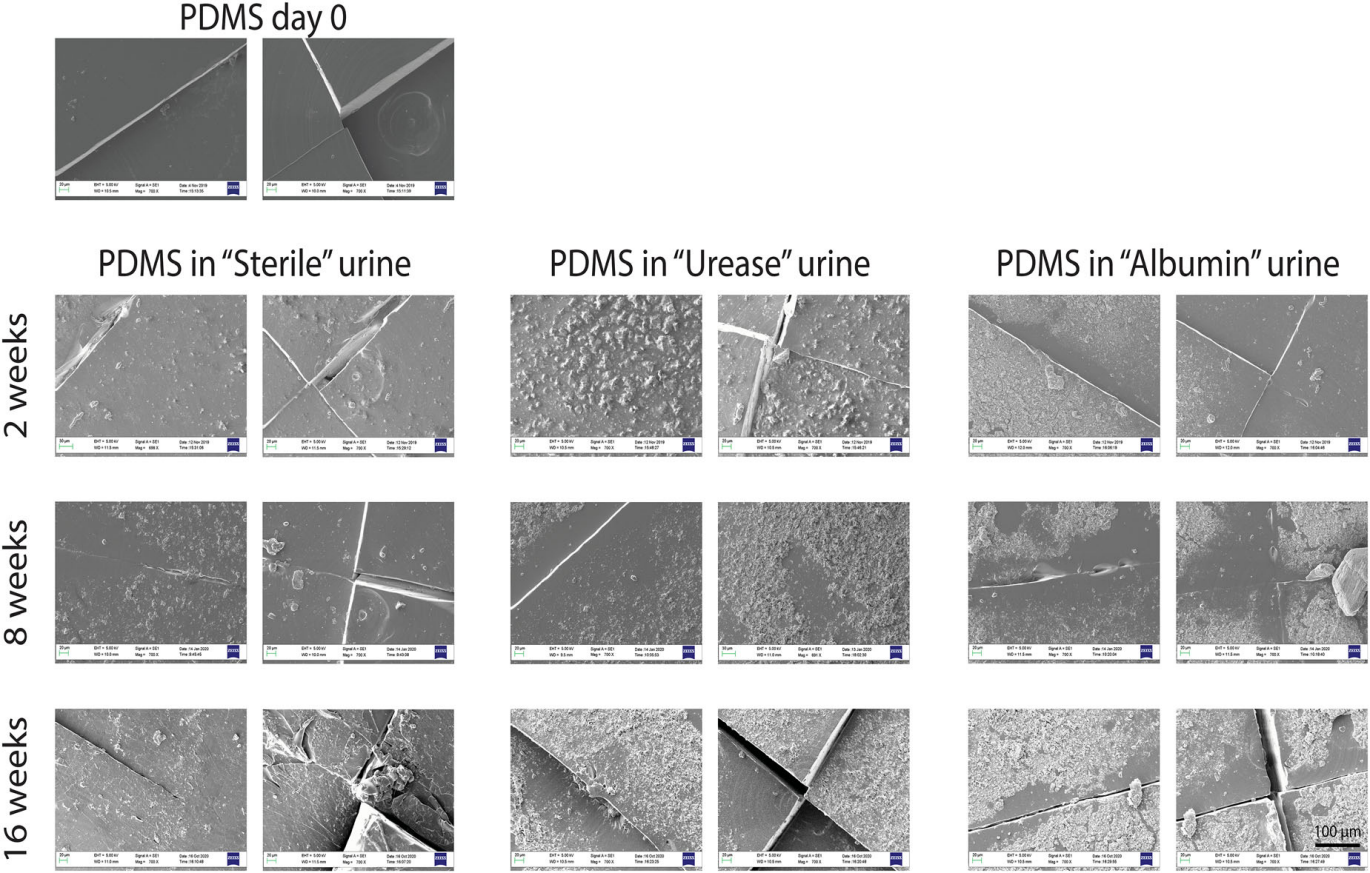
Urine test: SEM images (a zoom and image in the opening part). First, second, and third time‐points (2, 8, and 16 weeks) for PDMS in three types of artificial urine solutions: sterile (left), infected (middle), and infected + albumin (right). DLC, Diamond‐like carbon; PDMS, polydimethylsiloxane; SEM, scanning electron microscopy

**FIGURE 5:**
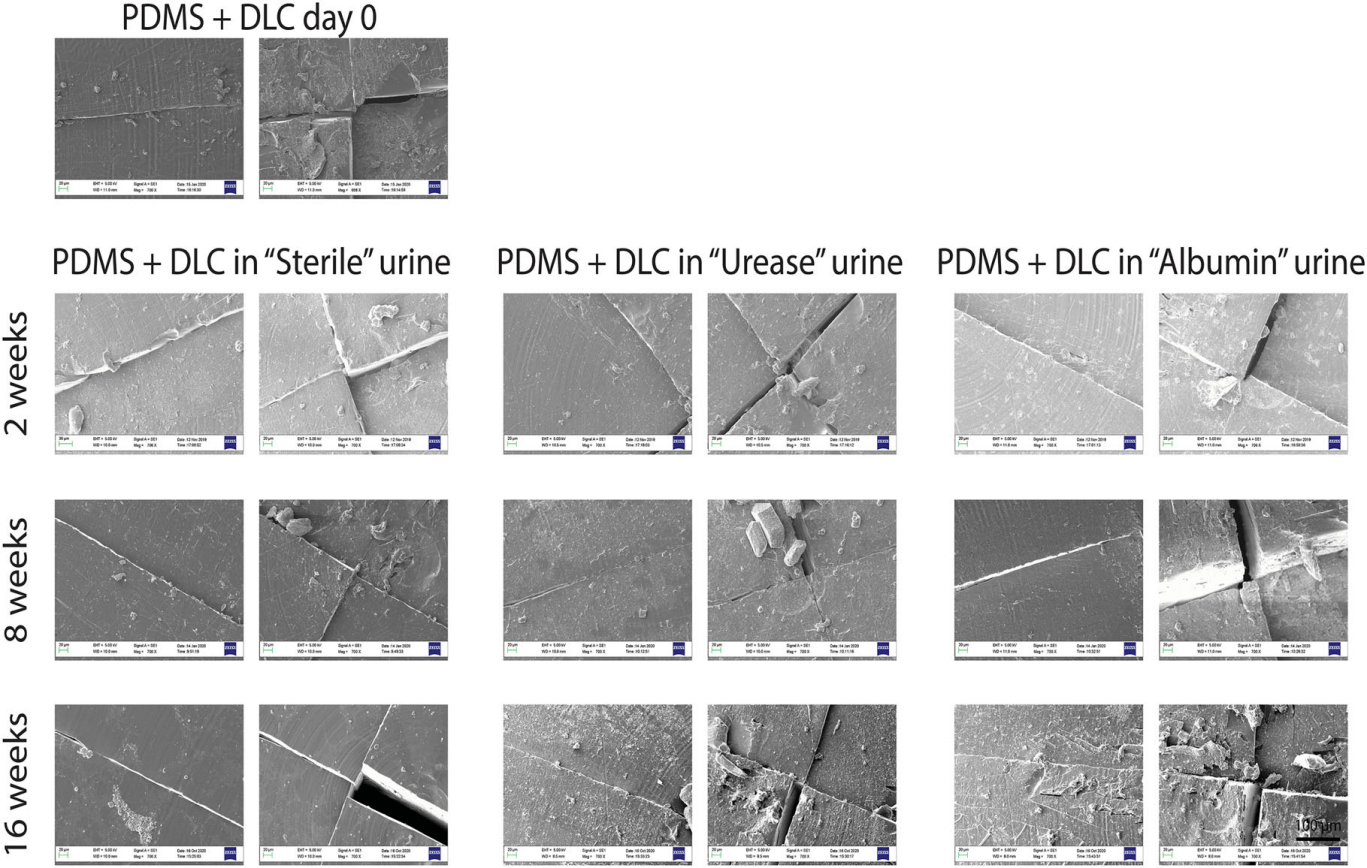
Urine test: SEM images. First, second, and third time‐points (2, 8, and 16 weeks) for PDMS + DLC valves in three types of artificial urine solutions: sterile (left), infected (middle), and infected + albumin (right). DLC, Diamond‐like carbon; PDMS, polydimethylsiloxane; SEM, scanning electron microscopy

Figure 4 shows the surface morphology obtained through SEM images of the different sample types for each time‐point of immersion in the different urine formulations and kept under continuous mechanical agitation at 37°C. In “Sterile” urine, even after 8 and 16 weeks, the presence of encrustations on the PDMS valves are rather scarce, while more encrustations were formed on valves immersed in “Urease” urine and in “Albumin” urine. However, such encrustations constituted a thin layer, without the formation of large brushite or struvite crystals. The valves provided with the DLC coating showed a similar behavior, but with even less crystals formed on top of them, showing more pronounced antifouling characteristics (Figure 5).

**FIGURE 6:**
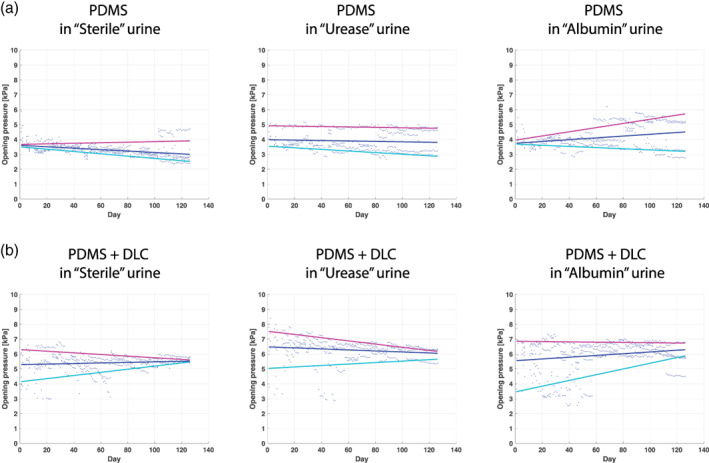
Urine test. Opening pressure versus time for (A) PDMS valves and (B) PDMS + DLC valves immersed in three types of artificial urine solutions: sterile (left), infected (middle), and infected + albumin (right). For each types of artificial urine, six valves in PDMS and six valves in PDMS + DLC were immersed for 126 days and tested five times per day with the setup shown in Figure 1H by flowing 200 ml of water. The blue points are the experimental data, the purple and the cyan lines, respectively, show the linear fitting of the maximum and the minimum value of the experimental data recorded each day while the blue line shows the linear fitting of the experimental data. DLC, Diamond‐like carbon; PDMS, polydimethylsiloxane

The analysis of the opening pressures over time showed that, for the PDMS valves, the overall functionality remained good in the considered time period: The opening pressure remained in fact below 6 kPa, which is the threshold for a suitable operation of an endo‐urethral artificial sphincter, in the three urine types, despite the thin layer of encrustations on the valve surface (Figure 6A). In the “Sterile” conditions, the opening pressure decreased over time of 0.6 kPa corresponding to a reduction of 16%. In the “Urease” condition, the opening pressure decreased by 2%. However, in the “Albumin” condition, the valve rigidity increased but, as mentioned, keeping its performance below the 6 kPa threshold. The average percentage variation in the opening pressure was 20%.

**FIGURE 7:**
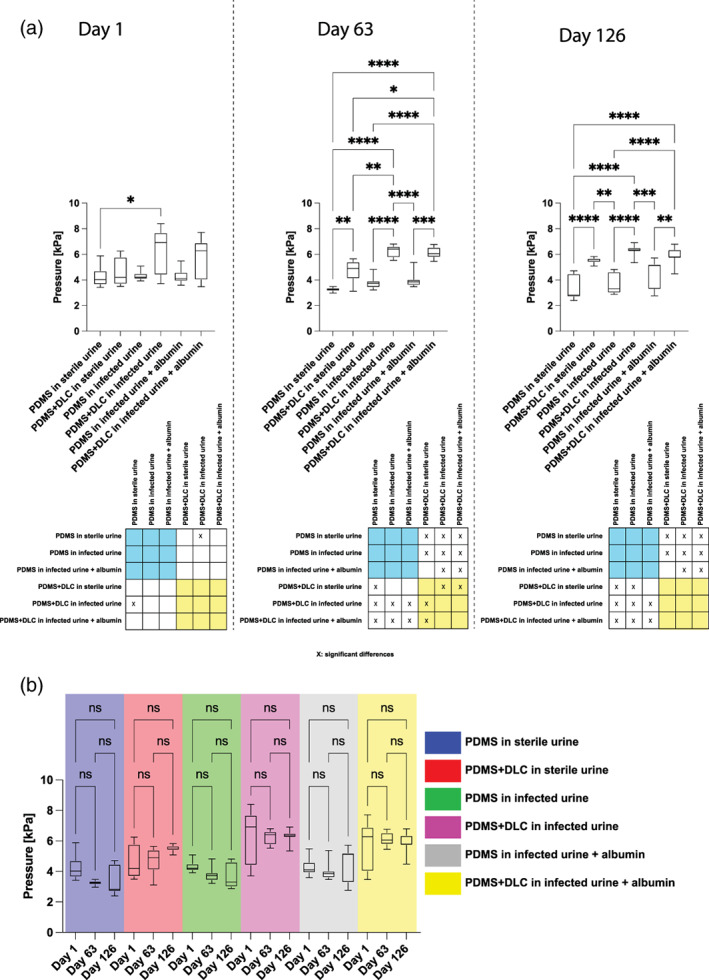
Comparative analysis: (A) Comparison between the two valve types (PDMS and PDMS + DLC) in the three urine formulations (“Sterile”, “Infected,” and “Infected + albumin”) at three time‐points at the bottom of (A) an overview table is shown in order to simplify the reading of the comparative analysis: The yellow box highlights the comparative analysis results between PDMS + DLC valves while the blue one highlights the comparative analysis results between the PDMS valves; (B) comparison at three time‐points of single valve types immersed into the respective urine formulation. Normality tests (Shapiro–Wilk) were performed on all experimental data to assess the data distribution, resulting in non‐normal ones. Results were expressed as median and 5–95 percentiles. Kruskal–Wallis with Dunn's post hoc tests were adopted for sample comparisons. Statistical analyses were carried out using GraphPad Prism (v 8.0.2). DLC, Diamond‐like carbon; PDMS, polydimethylsiloxane. The significance threshold was set at 5% (^*^ *p* < .05)

As regard the PDMS + DLC valves, the opening pressure in the “Sterile” condition showed a slight increase equal to 5% while in the “Urease” condition it decreased by 6%. In the “Albumin” condition, the opening pressure increased by 12%. Analyzing the average performance in terms of opening pressure of the PDMS valves and those coated with DLC, statistically significant differences were not found at Day 1. However, during the immersion test, statistically significant differences appeared between the two valve types, at Day 63 and Day 126, regardless of the type of urine (Figure 7A). No statistically significant differences were found in the performance of each type of valve over the three time‐points (Figure 7B).

## 3 DISCUSSION AND CONCLUSIONS

The functionalization procedure used to apply the DLC coating on the PDMS valve altered the surface properties of the material as follows: (i) the contact angle varied from 119.0 ± 0.2° to 108 ± 3° (Figure 1C,D, (ii) the surface roughness changed from 0.8 ± 0.4 μm to 2.7 ± 0.9 μm (Figure 1E,F, and (iii) the initial opening pressure did not change (Figure 1H), but the valves provided with a DLC coating showed higher variability in terms of opening pressure during the 4‐month fatigue cycles compared to bare PDMS valves, which however was reduced in the time due to elimination of the DLC coating.

The slight decrease in contact angle values due to the DLC coating confirms previous findings.^13^ Differently, the marked increase in surface roughness values does not reflect the results obtained in previous studies.^19^, ^20^ This can be due to the deposition method and the parameters used for depositing the DLC layer, which were slightly different from previous reports. Interestingly, the additional rigid DLC layer had no influence on the initial valve opening dynamics. The coating was stable even after numerous fatigue cycles, but presented a small degree of exfoliation and cracking, which in turn increased the average roughness of the surface reaching a value of 5.23 ± 3.2 μm. This is probably the reason for the higher variability in terms of opening pressure during the 4‐month fatigue cycles, for the PDMS + DLC valves. DLC coatings have been proposed in the cardiovascular field and claimed as possible functional coatings to improve the hemocompatibility of cardiac and aortic valves.^20^, ^21^, ^22^, ^23^ Such coatings did not interfere with the opening performance of the valves; however, the opening dynamics of cardiac/aortic valves are very different from the one featuring the polymeric valve proposed in this study and usable in artificial sphincters. Also, the long‐term stability of DLC coatings was assessed in previous studies,^24^ but in very different conditions (without any deformation of the polymeric structure below them). To the best of our knowledge, the results shown in this article demonstrate, for the first time, the role of a DLC coating in the opening performance of a polymeric valve with features suitable for application as a component of artificial sphincters, thus providing useful information to this purpose.

The valves were then tested in terms of resistance to urinary encrustations. Encrustations are solid elements that form due to salt saturation in aqueous solutions. Through the generation of a first crystalline nucleus, a cascade mechanism is activated, progressively facilitating the growth of new crystallization nuclei that lead to urinary concretions.^25^ Crystallization pathways can happen without or in the presence of bacteria, giving rise, respectively, to sterile or bacteria‐dependent crystallization.^26^ Sterile crystallization depends on the surface properties of materials in contact with urinary fluids: Large roughness, surface defects, high surface energy, and other features promote the nucleation process.^27^ Instead, in the case of bacteria‐dependent crystallization, a pH variation is induced by urease‐producing bacteria (e.g., *Proteus mirabilis*): This causes a decrease in solubility of the urine salts, which leads to an increase in the crystal precipitation.^28^ Furthermore, bacterial infections lead to the formation of biofilms on surfaces. These are characterized by defects that increase the surface roughness offering an optimal condition for nucleation and growth of urinary fouling.^11^, ^18^

In order to reduce encrustations and infections, various solutions have been considered.^29^, ^30^, ^31^, ^32^, ^33^, ^34^, ^35^, ^36^, ^37^, ^38^, ^39^ However, these solutions are not suitable for long‐term applications (over several months/years): In the first case, the bactericidal coatings are able to act for only a limited period of time, linked to inevitable depletion of the used agent, and in the second case, the used antibiofouling coatings are normally featured by a rather weak adsorption affinity to the substrate resulting in a low stability.^40^ Thus, the search for the “optimal coating” is still an open issue in the state‐of‐the‐art of urinary devices. However, this concerns static devices. No hints are available on the dynamics of encrustations and their role in modulating the performance (in terms of opening pressure) of dynamically moving urinary devices.

We found that, after 126 days of immersion in urine, PDMS showed some encrustations. However, they constituted only a rather thin layer of the polymer surface, without the formation of large brushite and wurtzite aggregates that were already documented on PDMS after 28 days of immersion.^13^ Furthermore, the performances (opening pressure) remained almost unvaried over time. DLC coatings also guaranteed a low level of encrustations and constant performance over time, although with a higher variability between samples.

Our results shed new light in the field of urinary devices based on moving polymeric elements. In fact, the performance invariance of the polymeric valves suggests a self‐cleaning behavior due to the daily opening: Most encrustations that form in the previous 24 h are probably removed by the mechanical action of the valve, whose leaflets shift down and the flux of urine cleans the surface, before the valve closes again. This marks a crucial difference with a stationary condition (with no valve opening), in which crystals with dimensions greater than 0.1 mm and organized in several layers form in less than 1 month (Figure S2). Results also highlighted that the formation of encrustations can be further reduced with a DLC coating. However, the valves provided with such a coating showed a higher variability in the opening pressure. Thus, a polymeric valve made of PDMS, even with no coatings, can be considered a suitable solution guaranteeing long‐term functionality in the urinary system, if it is opened at least once per day. This condition is a conservative one, considering endo‐urethral AUS as a possible clinical application. In fact, the typical urination frequency is five times per day, which would make the self‐cleaning effect even more effective. The results reported in this article can be useful not only for endo‐urethral AUS, but also for the design of other devices placed in the urinary system and in contact with urine (thus subjected to encrustations), based on movable elements (e.g., artificial bladders, flexible catheters).

## 4 EXPERIMENTAL SECTION

### 4.1 Fabrication of valves and coating

Polymeric valves were fabricated by exploiting dedicated molds. The molds were composed of three parts, provided with through holes, needed for aligning the parts, and closing the mold. To achieve the die cut on the valve surface, a proper frame (designed to constrain the polymeric valve) and a custom blade were used. Both cutting frame and molds were fabricated in INOX AISI 316 L by Ognibene srl (Bologna, Italy). The valve fabrication process was based on the following steps: (1) PDMS preparation: Monomer and curing agent were mixed with a 20:1 ratio, then the mixture was degassed for 20 min in a vacuum chamber; (2) PDMS casting: Once the mixture was well degassed, it was casted into the mold; (3) polymerization: After PDMS casting, the mold was closed and thermally treated at 70°C for 2 h, to allow full‐valve polymerization; and (4) valve extraction and cutting: The mold was opened and the polymeric valve was removed. It was then subjected to die‐cutting by the dedicated frame and custom blade.

PE‐CVD (T < 180°C) was first used to deposit a first soft DLC layer (with a relatively low number of sp^3^ bonds), aimed at improving the adhesion on PDMS substrates. Such a layer had a thickness of ∼0.05 μm. On top of this, a hard‐DLC layer (with a higher number of sp^3^ bonds, to guarantee chemical inertia) was fabricated using the same technique. The thickness of the second layer was also ∼0.05 μm. Such a double‐layer structure avoided an abrupt transition of elastic modulus between the substrate and the coating, thus preventing the formation of cracks that could compromise the long‐term resistance to urine.

### 4.2 Surface characterization

Contact angle measurements were carried out by means of an optical tensiometer (Attension, Biolin Scientific) using the sessile drop technique; 5 μl water droplets were deposited on PDMS and PDMS + DLC samples prior to image acquisition. Three independent samples were analyzed for each sample type. For each sample, measures were done in three different areas.

To assess surface roughness, a DCM 3D optical profilometer (Leica, Wetzlar, Germany) was used. The analyzed parameter was *Ra* that represents the arithmetic average height parameter defined as the average absolute deviation of the roughness irregularities from the mean line. The postprocessing workflow consisted of (i) spatial median filtering in order to equalize all data due to the roughness introduced by the waviness components which is defined as “the irregularities whose spacing is greater than the roughness sampling length” and to eliminate the noise components and (ii) Gaussian filtering (with a cutoff of 250 μm) to separate the waviness components from the real‐roughness components. All these operations were performed using the Leica Map software.

### 4.3 Opening pressure assessment

The valve opening pressures were measured through a custom setup (Figure 1G). The setup consisted of a fluidic system with continuous recirculation of distilled water thanks to a pump (RS Pro MG2000, RS Components Ltd., UK). The setup included a sensing system based on two pressure sensors (MPXHZ6400A, FreeScale Semiconductor Inc.) and one flow sensor (FCH‐midi‐POM, Biotech, Germany), managed by Arduino DUE (Arduino SRL, Monza, Italy) and a dedicated program developed in MATLAB environment (MathWorks, Apple Hill Drive, Massachusetts).

### 4.4 Fatigue test

Fatigue tests were performed by using the setup shown in Figure 1G. The valves (six samples for each type) were subjected to 1350 cycles of opening/closure, corresponding to a lifetime of 9 months, assuming five urinations per day. At each cycle, 200 ml of water was flushed through the valve, opening it and measuring the corresponding pressure generated. In addition to the measurements of the opening pressure, the surface morphology was also qualitatively assessed through optical profilometry (same instrument and parameters described above) and SEM (ZEISS EVO 10, Carl Zeiss, Oberkochen, Germany). Samples were analyzed under high vacuum conditions (pressure: 10^−5^ Pa). The magnifications used for stability tests vary in the range from 350× up to 700× at a voltage of 5 kV. Representative images were acquired from two different areas of the samples.

### 4.5 Experiments in artificial urine

Three urine formulations were developed to perform encrustation experiments: (i) sterile artificial urine (Sterile); (ii) artificial urine with the addition of the urease enzyme, to simulate the presence of a bacterial infection (Urease); and (iii) artificial urine with the addition of the urease enzyme and the albumin protein (Albumin) to evaluate the role of the protein component in the encrustation process. Many researchers, in fact, consider albumin to be a catalyst of the encrustation process, as it is the main component found in the organic matrix of crystals.

The “Sterile” formulation was prepared as reported by Lock.^18^ All chemicals were purchased from Sigma‐Aldrich (Saint Luis, Missouri), and distilled water was heated and kept at 37°C. The following chemicals were then added: 1.70 g/L ammonium phosphate dibasic, 0.24 g/L calcium chloride, 1.50 g/L creatine, 0.50 g/L magnesium chloride hexahydrate, 2.02 g/L potassium chloride, 2.02 g/L sodium sulfate, and 0.76 g/L urea. The pH was adjusted to 6 by adding hydrochloric acid (37 vol/vol %). The solution was filtered through a 0.22‐μm poly(ether sulfone) filter (Sartorius) and stored at 4°C to avoid the spontaneous precipitation of crystals. The “Urease” formulation was obtained by adding urease (3 mg/L) to the “Sterile” one. The “Albumin” formulation was obtained by adding bovine serum albumin (13 mg/L) to the “Urease” one.

Twelve PDMS valves and twelve PDMS + DLC valves were immersed in each of the three types of urine. They were kept under continuous mechanical agitation at 37°C and were subjected to five cycles of opening/closing per day by flowing 200 ml of water at each time. The opening pressures were measured every day during such opening/closure actions. At certain time‐points (2, 8, and 16 weeks), three samples underwent SEM imaging (and were thus excluded from the set of samples continuing the immersion in urine). Encrustations were analyzed through SEM imaging, using the same equipment and parameters described for the fatigue tests.

## Supporting information


**Appendix** **S1**: Supporting Information

## Data Availability

Data available on request from the authors.

## References

[1] Milsom I , Coyne KS , Nicholson S , Kvasz M , Chen C‐I , Wein AJ . Global prevalence and economic burden of urgency urinary incontinence: a systematic review. Eur Urol. 2014;65(1):79‐95. 24007713 10.1016/j.eururo.2013.08.031

[2] Irwin DE , Kopp ZS , Agatep B , Milsom I , Abrams P . Worldwide prevalence estimates of lower urinary tract symptoms, overactive bladder, urinary incontinence and bladder outlet obstruction. BJU Int. 2011;108(7):1132‐1138. 21231991 10.1111/j.1464-410X.2010.09993.x

[3] Riemsma R , Hagen S , Kirschner‐Hermanns R , et al. Can incontinence be cured? A systematic review of cure rates. BMC Med. 2017;15(1):63. 28335792 10.1186/s12916-017-0828-2 PMC5364653

[4] Global Incontinence Care Products and Devices Market 2021 with a Top Companies Data ‐ Size and Share, Trends Analysis, Growth, In‐depth Analysis for New Business Opportunities, Market‐Specific Challenges and Demands ‐ Forecast upto 2026.

[5] Marziale L , Lucarini G , Mazzocchi T , et al. Artificial sphincters to manage urinary incontinence: a review. Artif Organs. 2018;42(9):E215‐E233. 30074617 10.1111/aor.13164

[6] Hached S , Saadaoui Z , Loutochin O , Garon A , Corcos J , Sawan M . Novel, wirelessly controlled, and adaptive artificial urinary sphincter. IEEE ASME Trans Mechatron. 2015;20(6):3040‐3052.

[7] Lamraoui H , Bonvilain A , Robain G , et al. Development of a novel artificial urinary sphincter: a versatile automated device. IEEE ASME Trans Mechatron. 2010;15(6):916‐924.

[8] Miller JL , Bavendam T . Treatment with the reliance® urinary control insert: one‐year experience. J Endourol. 1996;10(3):287‐292. 8740394 10.1089/end.1996.10.287

[9] Elliott DS , Boone TB . Urethral devices for managing stress urinary incontinence. J Endourol. 2000;14(1):79‐83. 10735576 10.1089/end.2000.14.79

[10] Mazzocchi T et al. Magnetically controlled endo‐urethral artificial urinary sphincter. Ann Biomed Eng. 2017;45(5):1181‐1193. 28028713 10.1007/s10439-016-1784-2

[11] Shaw GL , Choong SK , Fry C . Encrustation of biomaterials in the urinary tract. Urol Res. 2005;33(1):17‐22. 15614579 10.1007/s00240-004-0423-9

[12] Mazzocchi T , Ricotti L , Pinzi N , Menciassi A . Parametric design, fabrication and validation of one‐way polymeric valves for artificial sphincters. Sens. Actuators, A. 2015;233:184‐194.

[13] Cardona A , Iacovacci V , Mazzocchi T , Menciassi A , Ricotti L . Novel nanostructured coating on PDMS substrates featuring high resistance to urine. ACS Appl. Bio Mater. 2018;2(1):255‐265. 10.1021/acsabm.8b00586 35016348

[14] Kleinen L , Syring I , Laube N . Reduction of biofilm formation on a‐C: H coated implants: investigation of biofilm‐surface interactions by variation of thin film properties. Plasma Processes Polym. 2009;6(S1):S41‐S45.

[15] Laube N , Kleinen L , Bradenahl J , Meissne A . Diamond‐like carbon coatings on ureteral stents—a new strategy for decreasing the formation of crystalline bacterial biofilms? J Urol. 2007;177(5):1923‐1927. 17437849 10.1016/j.juro.2007.01.016

[16] Jones DS , Garvin CP , Dowling D , Donnelly K , Gorman SP . Examination of surface properties and in vitro biological performance of amorphous diamond‐like carbon‐coated polyurethane. J Biomed Mater Res B Appl Biomater. 2006;78(2):230‐236. 16615067 10.1002/jbm.b.30474

[17] Fischer CB , Rohrbeck M , Zentgraf S , Wehner S . Diamond‐like carbon coatings on medically relevant polyurethane tubing with a follow‐up aging study. Chemistry. 2014;4:5.

[18] Lock JY , Wyatt E , Upadhyayula S , et al. Degradation and antibacterial properties of magnesium alloys in artificial urine for potential resorbable ureteral stent applications. J Biomed Mater Res A. 2014;102(3):781‐792. 23564415 10.1002/jbm.a.34741

[19] Ban M , Tobe S , Takeuchi L . Effects of diamond‐like carbon thin film and wrinkle microstructure on cell proliferation. Diamond Relat Mater. 2018;90:194‐201.

[20] Liu JQ , Li L , Wei B , Wen F , Cao H , Pei Y . Effect of sputtering pressure on the surface topography, structure, wettability and tribological performance of DLC films coated on rubber by magnetron sputtering. Surf Coat Technol. 2019;365:33‐40.

[21] Ali N , Kousa Y , Gracio J , et al. Surface engineering of artificial heart valves to using modified diamond‐like coatings. In: Jackson MJ , Ahmed W , eds. Surgical Tools and Medical Devices. Cham: Springer; 2016:117‐147.

[22] Woo, Yi‐ren et al. "Medical article with a diamond‐like carbon coated polymer." U.S. Patent No. 6,761,736. 13 Jul. 2004.

[23] Vallana, Franco , Pietro Arru & Marco Santi "Prosthesis of polymeric material coated with biocompatible carbon." U.S. Patent No. 5,370,684. 6 Dec. 1994.

[24] Cloutier M , Harnagea C , Hale PS , Seddiki O , Rosei F , Mantovani D . Long‐term stability of hydrogenated DLC coatings: effects of aging on the structural, chemical and mechanical properties. Diamond Relat Mater. 2014;48:65‐72.

[25] Kavanagh JP . Supersaturation and renal precipitation: the key to stone formation? Urol Res. 2006;34(2):81‐85. 16437224 10.1007/s00240-005-0015-3

[26] Balaji KC , Menon M . Mechanism of stone formation. Urol Clin North Am. 1997;24(1):1‐11. 9048848 10.1016/s0094-0143(05)70350-5

[27] Cox AJ . Comparison of catheter surface morphologies. Br J Urol. 1990;65(1):55‐60. 2310933 10.1111/j.1464-410x.1990.tb14662.x

[28] Choong S , Wood S , Fry C , Whitfield H . Catheter associated urinary tract infection and encrustation. Int J Antimicrob Agents. 2001;17(4):305‐310. 11295413 10.1016/s0924-8579(00)00348-4

[29] Lawson MKC , Shoemaker RK , Hoth KB , Bowman CN , Anseth K . Polymerizable vancomycin derivatives for bactericidal biomaterial surface modification: structure−function evaluation. Biomacromolecules. 2009;10(8):2221‐2234. 19606854 10.1021/bm900410a PMC2936007

[30] McCoy CP , Irwin NJ , Brady C , et al. An infection‐responsive approach to reduce bacterial adhesion in urinary biomaterials. Mol Pharm. 2016;13(8):2817‐2822. 27359363 10.1021/acs.molpharmaceut.6b00402

[31] Chuang HF , Smith RC , Hammond PT . Polyelectrolyte multilayers for tunable release of antibiotics. Biomacromolecules. 2008;9(6):1660‐1668. 18476743 10.1021/bm800185h

[32] Rai M , Yadav A , Gade A . Silver nanoparticles as a new generation of antimicrobials. Biotechnol Adv. 2009;27(1):76‐83. 18854209 10.1016/j.biotechadv.2008.09.002

[33] Wang R , Neoh KG , Kang E‐T , Tambyah PA , Chiong E . antifouling coating with controllable and sustained silver release for long‐term inhibition of infection and encrustation in urinary catheters. J Biomed Mater Res B Appl Biomater. 2015;103(3):519‐528. 24922113 10.1002/jbm.b.33230

[34] Bhargava A , Pareek V , Choudhury SR , Panwar J , Karmakar S . Superior bactericidal efficacy of fucose‐functionalized silver nanoparticles against Pseudomonas aeruginosa PAO1 and prevention of its colonization on urinary catheters. ACS Appl Mater Interfaces. 2018;10(35):29325‐29337. 30096228 10.1021/acsami.8b09475

[35] Krishnan S , Weinman CJ , Ober CK . Advances in polymers for anti‐biofouling surfaces. J Mater Chem. 2008;18(29):3405‐3413.

[36] Carlos DB , Ortner A , Dimitrov R , Navarro A , Mendoza E , Tzanov T . Building an antifouling zwitterionic coating on urinary catheters using an enzymatically triggered bottom‐up approach. ACS Appl Mater Interfaces. 2014;6(14):11385‐11393. 24955478 10.1021/am501961b

[37] Zhang Z , Vaisocherová H , Cheng G , Yang W , Xue H , Jiang S . Nonfouling behavior of polycarboxybetaine‐grafted surfaces: structural and environmental effects. Biomacromolecules. 2008;9(10):2686‐2692. 18785772 10.1021/bm800407r

[38] Keum H , Kim JY , Yu B , et al. Prevention of bacterial colonization on catheters by a one‐step coating process involving an antibiofouling polymer in water. ACS Appl Mater Interfaces. 2017;9(23):19736‐19745. 28569502 10.1021/acsami.7b06899

[39] Lalitha K , Miryala S , Yadavali SP , et al. Intrinsic hydrophobic antibacterial thin film from renewable resources: application in the development of anti‐biofilm urinary catheters. ACS Sustain. Chem. Eng. 2017;5(1):436‐449.

[40] Zhang H , Chiao M . Anti‐fouling coatings of poly (dimethylsiloxane) devices for biological and biomedical applications. J Med Biol Eng. 2015;35(2):143‐155. 25960703 10.1007/s40846-015-0029-4 PMC4414934

