# Supplementary material for: PDMS and DLC‐coated unidirectional valves for artificial urinary sphincters: Opening performance after 126 days of immersion in urine
Source: J Biomed Mater Res B Appl Biomater. 2021 Nov 2;110(4):817–27. doi: 10.1002/jbm.b.34961 (PMC9298115; doi:10.1002/jbm.b.34961)
Supplement: Supplementary file 1 — Appendix S1: Supporting Information [file JBM-110-817-s001.docx]

**Supporting Information**

**PDMS and DLC-coated unidirectional valves for artificial urinary sphincters: opening performance after 126 days of immersion in urine**

Tommaso Mazzocchi^1,2^, Gioia Lucarini^1,2^, Irene Roehrer^1,2^, Arianna Menciassi^1,2^, Leonardo Ricotti^1,2^.

^1^The BioRobotics Institute, Scuola Superiore Sant’Anna, Piazza Martiri della Libertà 33, 56127 Pisa, Italy

^2^Department of Excellence in Robotics & AI, Scuola Superiore Sant’Anna, Piazza Martiri della Libertà 33, 56127 Pisa, Italy

Corresponding Author

Gioia Lucarini

The BioRobotics Institute, Scuola Superiore Sant’Anna

Viale R. Piaggio 34, 56025 – Pontedera (PI), Italy

Tel: +39 050 883074 / Mobile: +39 366 6868242

e-mail: [g.lucarini@santannapisa.it](mailto:g.lucarini@santannapisa.it)

**
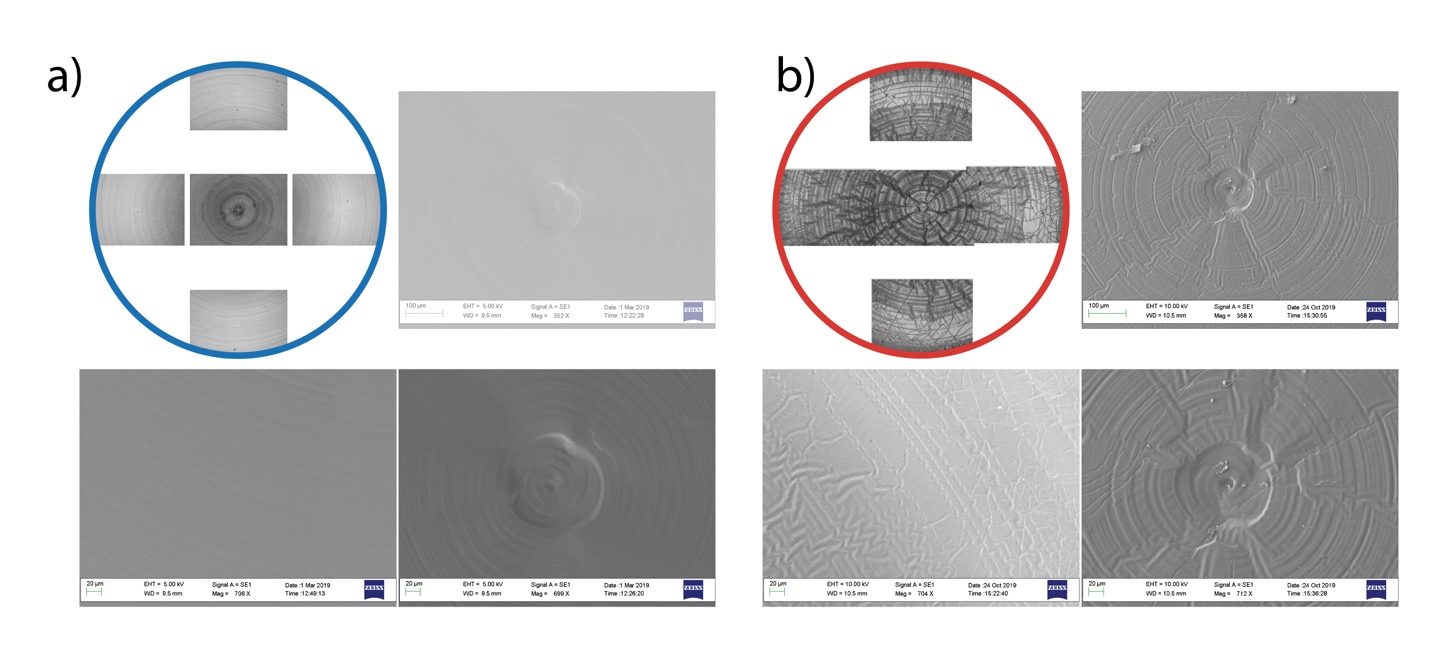
**

Figure S1 SEM images. Comparison between PDMS (a) and PDMS + DLC (b) valves before to apply the die cutting to the valve.

**
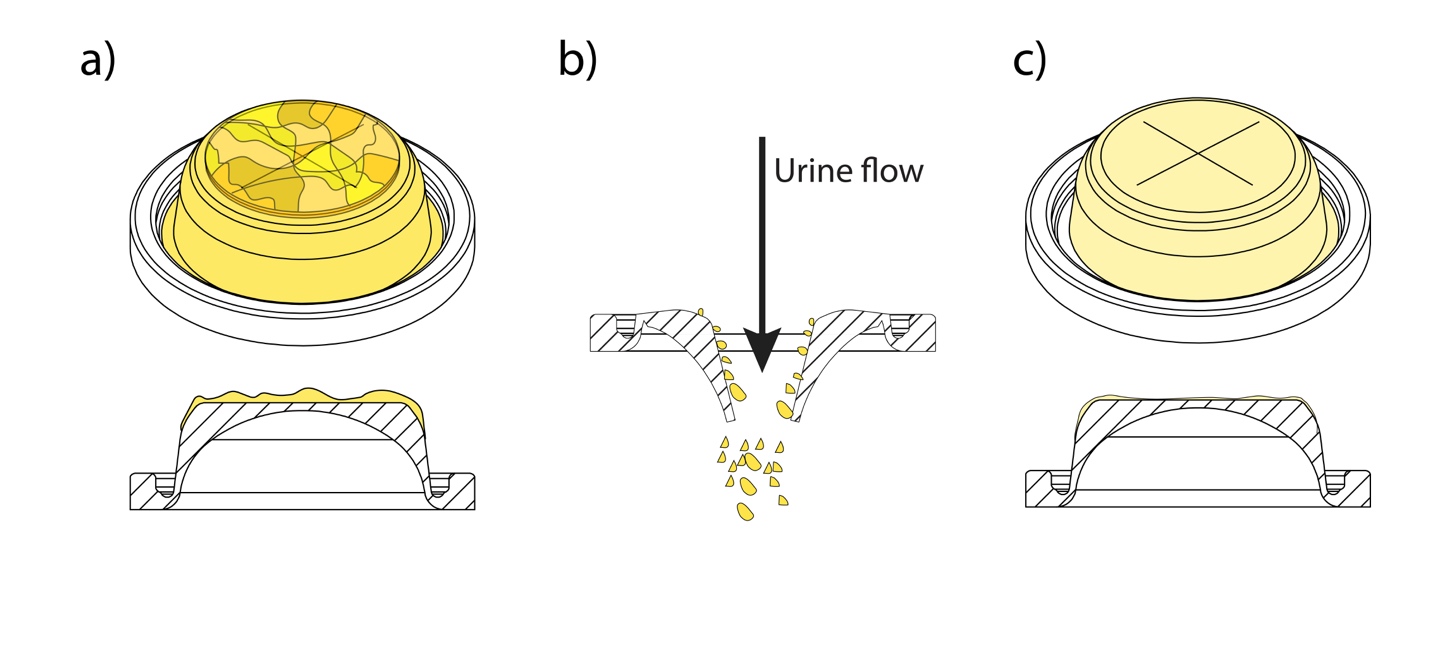
**

Figure S 2 Polymeric valves self-cleaning behavior. The contact of the urine with the polymer valve generates a layer of encrustations under static conditions (a). However when the polymer valve opens daily, the constrictions detach from the surface and are eliminated by the urine flow (b). At the end of urination, the encrustation layer is reduced in relation to the frequency of opening (c).
